# The Potential Role of miRNAs in Cognitive Frailty

**DOI:** 10.3389/fnagi.2021.763110

**Published:** 2021-11-12

**Authors:** Giulia Carini, Laura Musazzi, Francesco Bolzetta, Alberto Cester, Chiara Fiorentini, Alessandro Ieraci, Stefania Maggi, Maurizio Popoli, Nicola Veronese, Alessandro Barbon

**Affiliations:** ^1^Department of Molecular and Translational Medicine, University of Brescia, Brescia, Italy; ^2^School of Medicine and Surgery, University of Milano-Bicocca, Milan, Italy; ^3^Medical Department, Geriatric Unit, Azienda ULSS (Unità Locale Socio Sanitaria) 3 “Serenissima,” Venice, Italy; ^4^Department of Pharmaceutical Sciences, University of Milan, Milan, Italy; ^5^Aging Branch, Neuroscience Institute, National Research Council, Padua, Italy; ^6^Geriatrics Section, Department of Medicine, University of Palermo, Palermo, Italy

**Keywords:** frailty, cognitive frailty, biomarkers, miRNA–microRNA, cognitive impairment, MCI (mild cognitive impairment)

## Abstract

Frailty is an aging related condition, which has been defined as a state of enhanced vulnerability to stressors, leading to a limited capacity to meet homeostatic demands. Cognitive impairment is also frequent in older people, often accompanying frailty. Age is the main independent risk factor for both frailty and cognitive impairment, and compelling evidence suggests that similar age-associated mechanisms could underlie both clinical conditions. Accordingly, it has been suggested that frailty and cognitive impairment share common pathways, and some authors proposed “cognitive frailty” as a single complex phenotype. Nevertheless, so far, no clear common underlying pathways have been discovered for both conditions. microRNAs (miRNAs) have emerged as key fine-tuning regulators in most physiological processes, as well as pathological conditions. Importantly, miRNAs have been proposed as both peripheral biomarkers and potential molecular factors involved in physiological and pathological aging. In this review, we discuss the evidence linking changes of selected miRNAs expression with frailty and cognitive impairment. Overall, miR-92a-5p and miR-532-5p, as well as other miRNAs implicated in pathological aging, should be investigated as potential biomarkers (and putative molecular effectors) of cognitive frailty.

## Introduction

The greatest achievement of public healthcare in the last several decades has been the large increase in lifespan. Yet the increasing aging population has brought about new challenges to the health system, with the mounting prevalence of geriatric conditions requiring a new general healthcare system for people afflicted by physical and mental impairment (Beard et al., 2016; Howdon and Rice, 2018).

In older people, frailty and cognitive impairment are commonly found together (Fabricio et al., 2020). Frailty is a clinical syndrome with different definitions, generally referred as a state of increased vulnerability to stressors that results from a decreased physiological reserve in multiple organs and systems, leading to a limited capacity to meet homeostatic demands (Clegg et al., 2013; Proietti and Cesari, 2020). Although frailty and cognitive impairment could be considered as distinct clinical states, converging evidence has shown a close epidemiological association between these conditions (Halil et al., 2015; Kiiti Borges et al., 2019; Miyamura et al., 2019). This led to the generation of the term “cognitive frailty,” defined as a heterogeneous clinical condition characterized by the concomitant presence of both physical frailty and cognitive impairment (Kelaiditi et al., 2013).

**FIGURE 1:**
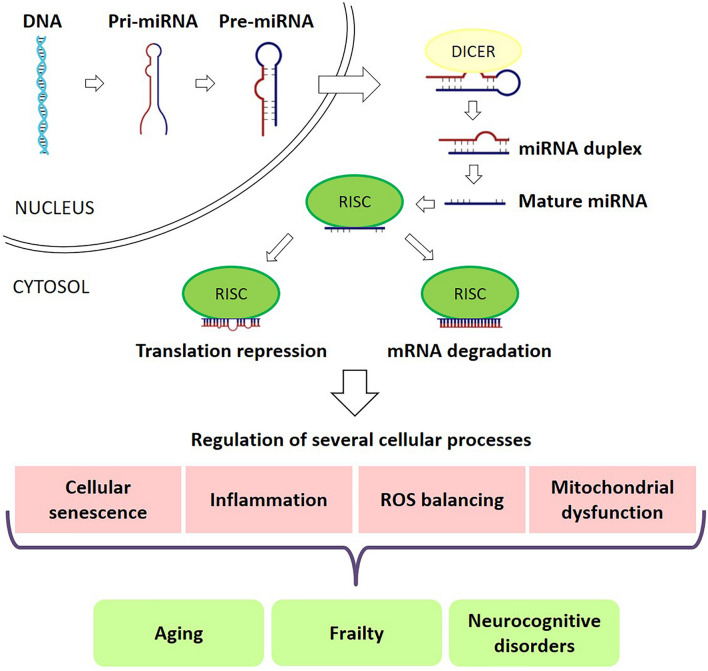
miRNAs in frailty and cognitive deficits. miRNAs play a major role in RNA silencing and post-transcriptional regulation of gene expression. miRNAs target hundreds of transcripts to regulate various biological pathways and processes, repressing translation or inducing mRNA degradation of target transcripts through sequence-specific binding. miRNAs are key modulators of almost all physiological processes and, consequently, miRNA dysregulation is seen in a multiplicity of diseases, including frailty and cognitive deficits.

Nevertheless, the molecular mechanisms underlying cognitive frailty are still largely unknown. microRNAs (miRNAs) are a large family of conserved small (20–22 nucleotides) non-coding RNAs involved in post-transcriptional regulation of gene expression. Each miRNA targets hundreds of transcripts mainly repressing translation or inducing mRNA degradation of target transcripts through sequence-specific binding (Mohr and Mott, 2015). Compelling evidence suggests that miRNAs are both involved in physiological/pathological processes associated with aging (Williams et al., 2017) and in the regulation of brain functions (Kumar et al., 2017a; Nampoothiri and Rajanikant, 2017). Indeed, miRNAs act in several biological functions, such as proliferation, apoptosis, cell differentiation, embryogenesis, organogenesis, signal transduction and metabolism (Alvarez-Garcia and Miska, 2005; Kloosterman and Plasterk, 2006). Thus, it should not be surprising that miRNAs were recognized as key modulators of virtually all physiological processes and, consequently, miRNAs dysregulation have been reported in a multiplicity of diseases (Figure 1; Condrat et al., 2020). In addition to their presence inside the cells, miRNAs can be found also in extracellular fluids, forming the so-called circulating miRNAs, which are supposed to be involved in cell signaling and communication (Sohel, 2016). miRNAs presence in body fluids can be due to several concomitant processes, including tissue damage, cell apoptosis and necrosis, active release in exosomes and microvesicles, or association with proteins (O’Brien et al., 2018).

In the present manuscript, after introducing the multiple clinical aspects and the main cellular mechanisms proposed to be associated with frailty and cognitive impairment, we designed a narrative review on the studies in which miRNAs have been proposed as peripheral circulating biomarkers for frailty or cognitive impairment, with the final aim to identify miRNAs that might be associated with cognitive frailty.

## Clinical, Cellular, and Molecular Mechanisms of Frailty: The Potential Role of miRNAs

### Clinical Features of Frailty

Frailty is generally considered as a geriatric syndrome, characterized by an excessive vulnerability to endogenous and exogenous stressors, due to a decrease in physiological reserves, thus leading to a high risk of developing adverse health outcomes (Clegg et al., 2013; Proietti and Cesari, 2020).

The majority of studies are based on the definition of frailty introduced by Fried and collaborators in 2001. The Fried phenotype also known as the frailty phenotype model defines frailty as a clinical syndrome in which three or more of the following criteria are present: unintentional weight loss, fatigue or self-reported exhaustion, weakness (poor grip strength), slow walking speed, and reduced or absent physical activity (Fried et al., 2001). This definition, exclusively considering the physical domain, is most frequently used for determining “physical frailty.” It should be mentioned that a key contribution to physical frailty comes from sarcopenia, defined as a progressive loss of skeletal muscle mass and strength (Ardeljan and Hurezeanu, 2021). Sarcopenia and frailty often co-exist in older patients, presenting a significant overlap of physical symptoms (Martin and Ranhoff, 2020). Indeed, sarcopenia is viewed as an essential correlate of the physical component of the frailty phenotype, although frailty can also be present in the absence of sarcopenia, suggesting the existence of several phenotypes of frailty (Davies et al., 2018).

In the same year in which Fried published the clinical criteria of physical frailty, other authors started to recognize that frailty was not exclusively characterized by physical impairments, but could be considered a more complex condition, involving other functional domains. Indeed, Rockwood and Mitnitski proposed the so-called Frailty Index (or Frailty Index of Deficit Accumulation) (Mitnitski et al., 2001), which is based on the concept that aging is a continuous process characterized by several deficits (including diseases, signs, symptoms, laboratory abnormalities, cognitive decline, and disabilities in activities of daily living), the accumulation of which may lead to frailty. Accordingly, the Frailty Index is defined as the proportion of accumulated deficits, thus representing the probability of an individual being frail (Martin and O’Halloran, 2020).

Other definitions of frailty exist, but the Fried Frailty Score and the Frailty Index are the most frequently used in clinical practice (Dent et al., 2016; Lekan et al., 2021).

More recently, a novel model of frailty has been proposed, based on a multidimensional evaluation considering the loss of harmonic interaction between multiple domains, including genetic, biological, functional, cognitive, psychological, and socio-economic dimensions, that ultimately leads to homeostatic instability (Pilotto et al., 2008, 2020). This multidimensional approach exploits the instruments of the comprehensive geriatric assessment (CGA). Operatively, CGA uses specific scales that explore functional disability, cognition, depression, nutritional status, comorbidities, number of drugs used, falls and pressure sores risk, cohabitation status, social and welfare context. This view, considering both multimorbidity and polypharmacy, allows for the evaluation of multidimensional impairment of the subject and promises to help the appropriateness of prescribing and intervention in frail older adults (Pilotto et al., 2018).

The prevalence of frailty has been assessed in many studies worldwide, although the results are highly variable, essentially depending on the definition used for indicating frailty. Overall, frailty has a prevalence estimated at around 11–16% in the population 60 years and older (Rohrmann, 2020; O’Caoimh et al., 2021). Frailty is more prevalent in women compared to men and as expected, prevalence increased with age, being the highest in subjects over 85 years (Collard et al., 2012; Rohrmann, 2020).

### Cellular and Molecular Mechanisms of Frailty

**FIGURE 2:**
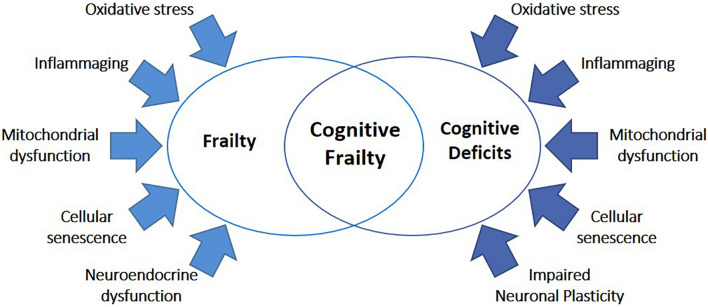
Common mechanisms underlying frailty and cognitive deficits. The high majority of mechanisms known to be involved in frailty were also implicated in cognitive diseases, including oxidative stress, inflammaging, mitochondrial dysfunction, cellular senescence, neuroendocrine dysfunctions, and impaired neuronal plasticity.

In the last years, great efforts have been made to discover the molecular mechanisms underlying frailty. A gradual decrease in physiological reserve occurs with physiological aging but, in frailty, this decrease is accelerated, and homeostatic mechanisms start to fail (Clegg et al., 2013). Although lifelong accumulation of molecular and cellular damages is believed as a key element of both physiological aging and frailty, the interplay among dysfunctions in the brain, endocrine system, immune system, and skeletal muscle functions is recognized as a main factor in the development of frailty (Figure 2; Clegg et al., 2013). In the following paragraphs, we resume the main systemic and cellular processes recognized to be involved in frailty pathophysiology, including changes in the immune system, cellular senescence, and hormonal imbalance.

#### Changes in the Immune System and Related Musculoskeletal Consequences

Aging is associated with dramatic changes in the immune system, implying both immunosenescence (the decline in immune function with aging), and inflammaging (a state of chronic inflammation), which are considered to be main risk factors for age-related diseases (Franceschi et al., 2000; Fulop et al., 2015, 2018). Immunosenescence is characterized by altered T and B cells responses due to a modified naïve/memory cell ratio. Accumulation of memory T cells and reduction of peripheral blood naïve T cells are observed as a result of developmentally programmed thymic involution, increased serum levels of IgG and IgA, and a poor response to newly encountered microbial antigens (Pawelec, 2018). On the other hand, inflammaging is characterized by increasing circulating pro-inflammatory factors and decreasing circulatory anti-inflammatory factors (Franceschi et al., 2018). Remarkably, frail people have both immunosenescence (Lang et al., 2010) and inflammaging (Soysal et al., 2016).

The immune system plays, directly and indirectly, a role in age-associated muscle decline. Multiple immune cells have been implicated in muscle repair and regeneration, by controlling the local inflammatory responses and promoting muscle growth through releasing growth factors (Xu et al., 2020). Moreover, inflammatory cytokines have a major role in muscle homeostasis, activating muscle breakdown to generate amino acids for energy and cleave antigenic peptides. However, the overactive, insufficiently regulated inflammatory response that characterizes aging and frailty could result in loss of muscle mass and strength, with an associated reduction in functional ability (Clegg et al., 2013; Wilson et al., 2017). Accordingly, as already mentioned in the introduction, sarcopenia is considered as a key component of frailty as well as a predictor of morbidity, disability, and death in older people (Cooper et al., 2012; Nascimento et al., 2018).

#### Cellular Senescence

Cellular repair and regeneration are key elements in tissue homeostasis (Lazzeri et al., 2012). Aging is characterized by the loss of tissue regenerative properties and the accumulation of senescent cells, which is a defense mechanism preventing genomic instability (Bisset and Howlett, 2019). Senescent cells are non-dividing cells, highly metabolically active, that gradually acquire a secretory phenotype called senescence-associated secretory phenotype (SASP) (Cardoso et al., 2018). SASP contains a variety of factors, including proinflammatory and matrix modifying peptides, which negatively influence tissue homeostasis and regeneration (Zampino et al., 2020) and are causally linked to increased inflammaging (Korolchuk et al., 2017). SASP has also been shown to be involved in the pathogenesis of several age-related diseases and conditions, including frailty (LeBrasseur et al., 2015; Schafer et al., 2020).

Senescence is associated with dysregulated mitophagy and mitochondrial dysfunction (Chapman et al., 2019), leading to enhanced levels of reactive oxygen species (ROS), which in turn contribute to the development of senescent phenotype (Korolchuk et al., 2017), age-related diseases, and frailty (El Assar et al., 2020; Ferrucci and Zampino, 2020).

#### Hormonal Imbalance

During aging, hormonal axes suffer significant changes. The endocrine system is considered particularly important in frailty, because of its complex inter-relationships with the brain, immune system, and skeletal muscle (Clegg and Hassan-Smith, 2018). Anabolic hormones, such as androgens and insulin-like growth factor-1 (IGF-1), play a key role in stimulating protein synthesis, muscle growth, and insulin secretion. Strong evidence suggested that the levels of these hormones decline with age (Bisset and Howlett, 2019) and their alteration have been associated with frailty (Morley and Malmstrom, 2013). Adrenocorticotropic Hormone (ACTH) and cortisol secretion are also altered during aging and frailty leading to an impaired ability to recover from stressful stimuli in older people (Yiallouris et al., 2019). The dysregulation of multiple hormones has been proposed as one potential mechanism underlying frailty since preliminary evidence indicates that the cumulative burden of hormone deficiencies in frailty may be more important than the type of hormonal change (Bisset and Howlett, 2019).

#### miRNAs and Frailty

miRNAs are emerging as promising non-invasive diagnostic and prognostic biomarkers, as well as potential therapeutic agents (Vatic et al., 2020). Indeed, they could be used both to help understand physiopathological processes, and as novel therapeutic strategies allowing the simultaneous targeting of different pathways (Cardoso et al., 2018).

The study of miRNAs is a growing area of interest in the aging field. miRNAs regulate several biological events related to the aging process but are also influenced by aging processes themselves (Figure 1). At the same time, miRNAs have been consistently linked with the main systemic and cellular processes discussed above as associated with frailty. Indeed, some miRNAs, defined as “inflamma-miRs,” are involved in inflammatory pathways modulation and are differentially expressed during inflammaging (Quinn and O’Neill, 2011; Boldin and Baltimore, 2012; Olivieri et al., 2013, 2017). miRNAs play a pivotal role also in sarcopenia, regulating different aspects of muscle homeostasis (Sannicandro et al., 2019; Kinser and Pincus, 2020; Yin et al., 2020). Moreover, other miRNAs, the so-called senescence-associated miRNAs (SA-miRs) are involved in crucial biological processes of cellular senescence such as apoptosis, mitochondrial metabolism, and mitochondrial dynamics (Bu et al., 2017; Geiger and Dalgaard, 2017; Suh, 2018).

Several studies have reported differential miRNA expression between young and older individuals without discriminating for a frail phenotype (ElSharawy et al., 2012; Olivieri et al., 2012; Serna et al., 2012; Noren Hooten et al., 2013; Smith-Vikos et al., 2016) reviewed in Chen et al. (2010) and Lai et al. (2019). Conversely, to the best of our knowledge, only two studies directly evaluated changes in blood plasma miRNAs in frailty (Table 1).

**TABLE 1:** Summary of miRNAs associated with frailty and cognitive deficits.

miRNAs associated with frailty				
Main findings	Participants	Sample	Technologies	Study
miR-10a-3p, **miR-92a-3p**, miR-185-3p, miR-194-5p, miR-326, **miR-532-5p**, miR-576-5p, miR-760	Seven young control subjects (30.3 ± 5.3), seven robust older subjects (76.0 ± 6.5), seven frail older subjects (85.6 ± 3.8)	Exosome isolated from the plasma	RNA-Seq	Ipson et al., 2018
miR-21	22 control subjects (20.5 ± 2.4), 34 aged robust subjects (76.6 ± 5.3), 40 aged fragile subjects (84.4 ± 5.6)	Plasma	qPCR	Rusanova et al., 2018
**miRNAs associated with cognitive impairment**				
**Main findings**	**Participants**	**Sample**	**Technologies**	**Study**
miR-7, miR-9, **miR-125b**, miR-127-3p, **mir-128, miR-132, miR-134,** miR-181a, **miR-323-3p, miR-382, miR-370, miR-491-5p, miR-874**	Pilot study: 10 control subjects (71-85), 10 MCI subjects (75-87). Main study: 20 young control subjects (21-50), 20 age matched control subjects (71-85), 20 MCI subjects (75-87), 20 AD patients (63-89). Longitudinal study: 19 subjects (73-84)	Plasma	qPCR	Sheinerman et al., 2012
**miR-128, miR-132, miR-134, miR-323-3p, miR-382, miR-370, miR-491-5p, miR-874**	50 control subjects (50-82), 20 MCI subjects (51-82)	Plasma	qPCR	Sheinerman et al., 2013
**miRNA-193b**	Age- and gender-matched control subjects, 43 MCI subjects (23 females, 20 males, 63.8 ± 6.1), 51 AD patients (28 females, 23 males, 64.2 ± 6.5)	Exosome isolated from the serum	qPCR	Liu et al., 2014a
**miR-384**	50 control subjects (28 females, 22 males, 63.9 ± 5.7 years), 32 MCI subjects (13 females, 19 males, 63.2 ± 6.1 years), 45 AD patients (18 females, 27 males, 64.2 ± 5.8 years)	Plasma, Serum	qPCR	Liu et al., 2014b
miR-200b	30 control subjects (75.2 ± 6.5), 32 MCI subjects (72.8 ± 6.1), 38 AD patients (76.2 ± 6.8)	Serum	qPCR	Liu et al., 2014c
**miR-93**, miR-143, miR-146a	123 control subjects (79.5 ± 6.8), 30 MCI subjects (81.1 ± 6.8), 127 AD patients (79.3 ± 8.9)	Serum	RNA-Seq qPCR validation	Dong et al., 2015
miR-107	81 control subjects (71.7 ± 5.4), 116 MCI subjects (68.6 ± 5.3), 97 AD patients (70.1 ± 4.6)	Plasma	qPCR	Wang et al., 2015
**miR-132, miR-206**	76 control subjects (73.17 ± 6.16), 66 MCI subjects (72.89 ± 7.59)	Serum	qPCR	Xie et al., 2015
**miR-210**	42 control subjects (23 males, 19 females, 62-85), 30 MCI subjects (18 males, 12 female patients, 61-82), 26 AD patients (12 males,14 females, 60-84)	Serum	qPCR	Zhu et al., 2015
miR-613	40 control subjects (22 females, 18 males, 63.2 ± 6.3), 32 MCI (22 females, 20 males, 64.8 ± 7.2), 48 AD patients (26 females, 22 males, 65.5 ± 6.8)	Serum	qPCR	Li et al., 2016
miR-101, **miR-103, miR-125b**, miR-191, miR-222	30 control subjects (70.4), 23 MCI patients (72.8)	Plasma	miRNA qPCR array	Kayano et al., 2016
miR-455-3p, miR-4668-5p	14 control subjects, 16 MCI subjects, 10 AD patients	Serum	miRNA array qPCR validation	Kumar et al., 2017b
miR-30b-5p, miR-142-3p, miR-200a-3p, miR-483-5p, miR-486-5p, miR-502-3p	Pilot Study: six control subjects (66 ± 5), seven MCI subjects (64.3 ± 6), seven AD patients (73.7 ± 5). Main Study: nine control subjects (66 ± 3), eight MCI subjects (65.8 ± 7), 13 AD patients (67.5 ± 8)	Plasma	miRNA qPCR array qPCR validation	Nagaraj et al., 2017
miR-135a, **miR-193b, miR-384**	Age- and gender-matched control subjects, 101 MCI subjects (59 females, 42 males, 61.63 ± 7.32), 107 AD patients (66 females, 41 males, 74.15 ± 7.93)	Exosome isolated from the serum	qPCR	Yang et al., 2018
miR-16-5p, **miR-92a-3p**, miR-26b-5p, miR-106b-5p, **miR-93-5p, miR-20a-5p**, miR-320a, let-7a-5p, miR-484, miR-615-3p, miR-18a-3p 5, miR-7977, miR-17-5p, miR-155-5p, **miR-193b-3p**, miR-450a-1-3p, miR-887-5p	GSE63063: Cohort 1: 104 control subjects (65 +); 80 MCI subjects (65 +), 142 AD patients (65 +). Cohort 2: 136 control subjects (65 +), 109 MCI subjects (65 +), 139 AD patients (65 +). GSE97760: 10 healthy controls (females, 72.1 ± 13.1), nine AD patients (females, 79.3 ± 12.3). E-MTAB-6094: 13 control subjects (10 females, three males, 77.3 ± 6.2), 22 AD patients (14 females, eight males, 79.4 ± 6.6)	Blood	Meta-Analysis of microarray data	Bottero and Potashkin, 2019
**miR-206**, miR-let-7b	Discovery cohort: 31 control subjects (75.0 ± 4.7), 30 MCI subjects (76.8 ± 4.0), 25 AD patients (84.6 ± 3.5). Longitudinal cohort: six control subjects (74.0 ± 3.2), six MCI to dementia subjects (77.3 ± 3.8), six stable MCI subjects (75.8 ± 3.6)	Plasma	miRNA qPCR array qPCR validation	Kenny et al., 2019
**miR-20a**, miR-27a, **miR-103a**	215 control subjects (138 females, 77 males, 60.9 ± 9.9), 122 lower SMMSE score subjects (55 females, 67 males, 67.6 ± 9.7)	Serum	qPCR	Kondo et al., 2019
**miR-92a-3p**, miR-181c-5p and **miR-210-3p**	14 control subjects (seven females, seven males, 68.29 ± 8.99), 26 MCI subjects (16 females, 10 males, 72.0 ± 8.49), 56 AD patients (41 females, 15 males, 77.77 ± 6.69),	Plasma	qPCR	Siedlecki-Wullich et al., 2019
miR-140-5p, miR-197-3p, miR-501-3p, miR-425-5p, **miR-532-5p**, miR-378a-5p, miR-411-3p, miR-181c-3p, miR-497-5p, miR-214-3p	94 control subjects (71.79 ± 9.46), 21 MoCA < 23 score subjects (72.29 ± 2.76)	Plasma	RNA-Seq	Gullett et al., 2020
miR-6764-5p, miR-6734-3p	Discovery cohort GSE120584: 288 control subjects (age 71.7 ± 6.3 years, 151 males and 137 females), 32 MCI subjects (age 75.5 ± 6.3 years, seven males and 25 females), 1,021 AD patients (age 79.2 ± 6.1 years, 307 males and 714 females). Validation cohort: four control subjects, five MCI subjects, six AD patients	Serum/Blood	Meta-Analysis of microarray data qPCR validation	Qin et al., 2021

*miRNAs in red were found in at least one frailty study and one study assessing cognitive function and miRNAs in blue were found in at least two studies assessing cognitive function.*

*The participants column shows the demographic characteristics of the subjects included in the study in accordance with the data available in the cited works (mean age, mean age ± SD, min–max age).*

*MCI, mild cognitive impairment; AD, Alzheimer’s disease; SMMSE, short Mini-Mental State Examination; MoCA score, Montreal Cognitive Assessment score.*

Ipson and collaborators examined the changes of plasma-derived exosome miRNA profiles in frailty, comparing young, old robust, and frail individuals. They identified eight miRNAs that were enriched in frailty: miR-10a-3p, miR-92a-3p, miR-185-3p, miR-194-5p, miR-326, miR-532-5p, miR-576-5p, and miR-760 (Ipson et al., 2018). The second study evaluated the levels of three inflammation-related miRNAs (miR-21, miR-146a, and miR-223) and one miRNA related to the control of melatonin synthesis (miR-483) in plasma samples of healthy adults, older robust, and frail patients. Frail subjects had higher miR-21 levels than controls, whereas miR-223 and miR-483 levels increased in both aged groups (Rusanova et al., 2018).

Although very preliminary, these two studies identified possible novel candidate biomarkers for frailty in old age. Intriguingly, some of these miRNAs were also related to cellular mechanisms involved in frailty pathogenesis. For example, miR-21 is counted among inflamma-miRs and is known to target a variety of molecules belonging to the NF-κB/NLRP3 pathways, thus modulating the “switch on/off of inflammation (Olivieri et al., 2013, 2021). miR-10a has been involved in inflammation as well (Tahamtan et al., 2018), while expression of miR-185-3p, miR-194-5p, and miR-760 have been associated with cellular senescence and ROS production (Lee et al., 2014; Bu et al., 2017; Xu et al., 2017; Suh, 2018; Li et al., 2020; Zhang et al., 2021). miR-194-5p and miR-92a-3p were reported to regulate muscle cell homeostasis (Morton et al., 2021; Shi et al., 2021a).

Moreover, some of these frailty-related miRNAs seem to play a major role also in neurons. Indeed, miR-326 inhibits neuronal apoptosis and attenuates mitochondrial damage (He et al., 2020; Huang et al., 2021). miR-532-5p showed a neuroprotective effect reducing apoptosis, ROS production, and inflammation in cerebral ischemia-reperfusion injury (Shi et al., 2021b), and ischemic stroke (Mu et al., 2020), while mir-92a-3p, belonging to the miR-17–92 family, is a synaptic-related miRNA (Siedlecki-Wullich et al., 2021), involved in neural cells proliferation, differentiation, and maturation (Zhang et al., 2013; Xia et al., 2020).

## Cognitive Impairment: The Potential Role of miRNAs

### Clinical Features of Cognitive Impairment

As we age, some cognitive abilities, such as language, vocabulary, and verbal skills, remain largely unchanged but other abilities, such as conceptual reasoning, memory, and processing speed, can physiologically decline gradually over time (Harada et al., 2013). Although general knowledge and crystallized intelligence are mostly unaffected during aging, fluid intelligence, which is the ability to learn and use new information and use it to problem-solve, is more affected (Deary et al., 2009).

Cognitive disorders are a general umbrella term that describes a group of conditions characterized by impairment in cognitive abilities such as memory, problem solving, and perception (Sachdev et al., 2013). Cognitive abilities are usually assessed through the administration of specific tests, i.e., the mini-mental state examination (MMSE) (Folstein et al., 1975) and the Montreal Cognitive Assessment (MoCA) (Nasreddine et al., 2005). Among cognitive disorders, mild cognitive impairment (MCI) is increasing in attention by researchers, as demonstrated by the introduction in the DSM-5. This entity can be identified in presence of: (1) modest cognitive decline from a previous level of performance in one or more cognitive domains, greater than expected for age, without falling into the dementia range, (2) no interference with capacity for independence in everyday activities, (3) cognitive deficits not occurring exclusively in the context of a delirium, and (4) cognitive deficits not explained by another mental disorder (Ganguli, 2013; Sachdev et al., 2013). MCI affects about 3–22% of the population over the age of 65 (Stokin et al., 2015; Sanford, 2017), symptoms may remain stable for years, with some cases may revert to normality (Sachdev et al., 2013), but it is estimated that about 50% of people affected by MCI can progress to dementia, particularly Alzheimer’s disease (AD) (Gordon and Martin, 2013).

### Cellular and Molecular Mechanisms Underlying Cognitive Impairment

Brain aging is the main predisposing factor for cognitive impairment (Yankner et al., 2008). As for frailty, the main mechanisms involved in cognitive disorders are also implicated in physiological aging (Figure 2). However, while a decline in cognitive features is expected during physiological aging, differently from pathological aging associated with cognitive decline, this does not result in any significant functional impairment (Schirinzi et al., 2020).

The pathophysiological mechanisms of cognitive disorders essentially comprise alterations of synaptic transmission, oxidative stress, cellular senescence, and increased inflammation.

#### Alterations of Synaptic Function

The maintenance of synaptic function requires the preservation of the proper synaptic structure, coordination of synaptic vesicle release and membrane excitability, and integration of retrograde signals from the postsynaptic terminal (Azpurua and Eaton, 2015). Aging is associated with physiological structural changes in the brain, including the reduction of the number and function of synapses in brain areas related to learning and memory (Burke and Barnes, 2006; Lupien et al., 2009; Cuestas Torres and Cardenas, 2020). However, beyond the physiological aging processes, more generalized synaptic deficits can induce cognitive disorders. The study of cellular mechanisms underlying cognitive impairment highlighted the role of synaptic dysfunction and synaptopathy, defined as an alteration of synaptic homeostasis leading to a high risk of degeneration and synaptic loss (Stephan et al., 2012; Skaper et al., 2017). Pathological changes identified in synaptic dysfunction include plaque and tangle formation, vascular pathologies, neurochemical deficits, cellular injury, oxidative stress, mitochondrial changes, inflammation, changes in genomic activity, disturbed protein metabolism (Stephan et al., 2012).

#### Oxidative Stress and Cellular Senescence

Neurons are postmitotic polarized cells with significant energy demands and mitochondria play a pivotal role in generating the ATP required to support electrochemical neurotransmission, synaptic plasticity, neural cell maintenance, and repair (Lejri et al., 2019). Defects in mitochondrial dynamics and quality control, together with inefficient mitochondrial transport and distribution in synaptic compartments, have been implicated in synaptic/neuronal degeneration and brain aging (Grimm and Eckert, 2017; Raefsky and Mattson, 2017). Apart from the production of energy, mitochondria are key modulators of brain cell survival and death by controlling calcium and redox equilibrium, producing ROS, and controlling cell apoptosis (Mattson and Arumugam, 2018). Cellular, biochemical, and molecular studies showed a clear link between oxidative stress and cognitive dysfunction during aging and age-associated neuronal diseases (Kandlur et al., 2020). Neurons are particularly vulnerable to oxidative insults: ROS may induce the activation of neuroinflammation and neuronal death, with mechanisms involving glutamate excitotoxicity, aspartate receptor signaling, and glucocorticoid receptor activation (Grimm and Eckert, 2017). Oxidative injury can alter brain plasticity, cell proliferation, neurogenesis, and synaptic neurotransmission while enhancing neuronal death and impairing normal synaptic neurotransmission (Castelli et al., 2019). Moreover, mitochondrial dysfunctions and ROS production trigger cell senescence of neurons and glial cells, which in turn contributes to changes in morphological and functional alterations associated with synaptopathy (Morley, 2018; Toricelli et al., 2021). Indeed, senescent cells secrete pro-inflammatory SASP factors and disrupt the cell-cell contacts needed for the structural and functional neuron–glial interaction that maintains neuronal homeostasis (Chinta et al., 2015).

#### Inflammation

The central nervous system is traditionally thought of as an immunologically privileged space, isolated from the immune system, and separated from peripheral immune cells that are unable to cross the blood-brain barrier. However, it is now accepted that there is a wide and constant bidirectional communication between the peripheral immune system and the central nervous system (Engelhardt et al., 2017). Indeed, it has been demonstrated that signals from a systemic inflammatory condition may contribute to brain immune cell population activation, which in turn may accelerate neuronal degeneration and/or cognitive decline, leading to exacerbation of a clinical condition (Perry, 2004). Although neuroinflammation serves several fundamental roles in the brain structure and function, chronic inflammation may instead cause an exaggerated response (Tangestani Fard and Stough, 2019). Resident glial cells, including microglia and astrocytes, become hyperactivated in response to inflammatory stimuli and sustain a high-level production of proinflammatory cytokines, chemokines, secondary messengers, and ROS (Shabab et al., 2017; Slota and Booth, 2019). This altered inflammatory status may contribute to the onset of cognitive impairment in older people and enhances the state of vulnerability to environmental challenges (Brivio et al., 2019).

### miRNAs and Cognitive Impairment

miRNAs have been shown to play a major role in the brain as key regulators of neuronal development from neural progenitor cells, cell migration, neuronal polarization, and synapse formation (Nampoothiri and Rajanikant, 2017; Rajman and Schratt, 2017; Esteves et al., 2020). miRNAs can also modulate neuroinflammation (Thounaojam et al., 2013; Sarkar et al., 2019; Slota and Booth, 2019), formation of ROS, mitochondrial function, and cellular senescence (Bigagli et al., 2016; Konovalova et al., 2019; Catanesi et al., 2020; Figure 1). Accordingly, it has been suggested that cognitive dysfunctions in aging may be predicted by selected alterations of miRNAs expression (Danka Mohammed et al., 2017; Hernandez-Rapp et al., 2017). Recently, the involvement of miRNAs in cognitive disorders has been extensively studied, measuring their levels in different body fluids, such as plasma, serum, urine, and cerebrospinal fluid (Grasso et al., 2014; Basak et al., 2016).

Changes in miRNA expression have been correlated with cognitive performance and decline.

Kondo and collaborators examined the association between cognitive function and serum levels of six miRNAs (miR-let-7d, miR-17, miR-20a, miR-27a, miR-34a, miR-103a) in 337 Japanese subjects who had never been diagnosed with dementia. This study identified a positive correlation between the serum levels of miR-20a, miR-27a, and miR-103a and MMSE scores. Thus, low serum miR-20a, miR-27a, and miR-103a levels were significantly associated with cognitive deficits and were proposed as markers of early-stage cognitive decline (Kondo et al., 2019).

A recent study utilized machine learning approaches as a broad cognitive screening instrument to determine whether miRNAs could be proposed as blood-based biomarkers of cognitive aging (Gullett et al., 2020). Top 10 most important miRNAs for predicting total cognitive performance include miR-140-5p, miR-197-3p, miR-501-3p, miR-425-5p, miR-532-5p, miR-378a-5p, miR-411-3p, miR-181c-3p, miR-497-5p, miR-214-3p. Instead, three miRNAs (miR-140-5p, miR-197-3p, miR-501-3p) were top-ranked predictors of multiple cognitive outcomes (including fluid, crystallized, and overall cognition).

Furthermore, several studies addressed alterations of miRNA profiles in the blood of MCI patients and proposed miRNAs as specific diagnostic and/or prognostic biomarkers of MCI (reviewed in Piscopo et al., 2019). Overall, more than forty miRNAs were reported to discriminate between MCI and healthy controls in different studies, although only miR-206 was consistently found as differentially expressed in at least two reports (Piscopo et al., 2019). Specific studies on MCI patients are reported in Table 1.

Moreover, a recent meta-analysis of six microarray datasets identified 17 miRNAs as dysregulated in both MCI and AD [miR-16-5p, miR-92a-3p, miR-26b-5p, miR-106b-5p, miR-93-5p, miR-20a-5p, miR-320a, let-7a-5p, miR-484, miR-615-3p,miR-18a-3p 5, miR-7977, miR-17-5p, miR-155-5p, miR-193b-3p, miR-450a-1-3p, miR-887-5p, suggesting a key involvement in the modulation of cognitive function (Bottero and Potashkin, 2019)].

Other miRNAs were instead proposed as early biomarkers of MCI in the preclinical stage, or for prodromal AD. miRNA pairs in the miR-132 family (miR-128/miR-491-5p, miR-132/miR-491-5p, and mir-874/miR-491-5p) and the miR-134 family (miR-134/miR-370, miR-323-3p/miR-370, and miR-382/miR-370), although not differentiating MCI from AD, were proposed as predictive markers for the onset of MCI (Sheinerman et al., 2012, 2013). On the other hand, Kenny and collaborators, based on a 4-year longitudinal evaluation, found increased miR-206 levels in MCI patients at high risk of dementia (tested with the Clinical Dementia Rating, CDR) and in MCI patients with deteriorating MMSE scores. Indeed, stable MCI subjects displayed little to no change in expression over the years, while MCI patients who progressed toward dementia displayed significantly higher levels of miR-206 (Kenny et al., 2019). Moreover, while upregulation of miR-92a, miR-181c, and miR-210 levels was reported in plasma of both MCI and AD patients, the signature values in the plasma of the MCI patients that progressed to AD were found to be significantly higher than the values found in the MCI patients that did not progress to dementia (Siedlecki-Wullich et al., 2019). Altogether, these data suggest that plasma levels of miR-206, miR-92a-3p, miR-181c-5p, and miR-210-3p could be used as molecular signatures of AD progression in MCI. Finally, very recently, Qin and collaborators, identified two miRNAs, miR-6764-5p and miR-6734-3p, as remarkably upregulated in both MCI and AD subjects compared to controls (Qin et al., 2021).

miRNAs reported in at least two studies as associated with cognitive function are highlighted in blue in Table 1.

## Cognitive Frailty: The Potential Role of miRNAs

### Cognitive Frailty: Definitions

Several shreds of evidence demonstrated that frailty and cognitive impairment are intrinsically related, since frailty is known to increase risk of cognitive decline, and cognitive decline may increase risk of frailty and have an impact on the trajectory of frailty (as recent reviews see Kiiti Borges et al., 2019; Welstead et al., 2020; Bu et al., 2021). The concept of simultaneous presence of frailty and cognitive impairment or cognitive frailty was initially proposed in 2013 by the International Institute of Nutrition and Aging and the International Geriatrics Association (IANA), defined by the presence of physical frailty and cognitive impairment, and exclusion of concurrent dementia (Kelaiditi et al., 2013). Although the concept of cognitive frailty is well accepted and has been shown to be associated with poor outcomes, there is yet no consensus on the actual definition (Merchant et al., 2021). Indeed, multiple definitions and terminologies have been proposed, including Motoric Cognitive Risk Syndrome (MCR), defined as presence of both slow gait speed and subjective cognitive complaints and absence of concurrent dementia or mobility disability (Verghese et al., 2014), or Physio-cognitive Decline Syndrome (PCDS), defined by slowness and/or weakness and ≥ 1.5 SD below age/sex/education-matched norms in any cognitive function domain (Chen and Arai, 2020). Moreover, Ruan and collaborators proposed a new classification of cognitive frailty, in which they distinguish “reversible” from “potential reversible” cognitive frailty. Reversible cognitive frailty was defined by the presence of physical/pre-physical frailty and subjective cognitive decline and/or positive fluid and imaging biomarkers of amyloid accumulation and neurodegeneration, while potentially reversible cognitive frailty was defined by the presence of physical/pre-physical frailty and cognitive impairment (Ruan et al., 2015).

Nevertheless, recent evidence suggests that, regardless of the specific definition, cognitive frailty is a target for preventing disability and dementia through multi-domain interventions, considering physical, nutritional, cognitive as well as psychological domains, with the final aim to modify the trajectory of frailty and cognitive decline toward positive outcomes.

Even though epidemiological and clinical studies have demonstrated a close relationship between frailty and cognitive diseases, the common/concurring molecular mechanisms are still largely unknown. Nevertheless, it has been proposed that abnormalities in biological processes related to physiological aging could play a major role in both conditions (Ruan et al., 2015; Searle and Rockwood, 2015). In particular, chronic inflammation, immunosenescence, imbalanced energy metabolism, mitochondrial dysfunction, oxidative stress, and neuroendocrine dysfunctions may be all involved in cognitive frailty (Figure 2; Mulero et al., 2011; Robertson et al., 2013; Fulop et al., 2018; Sargent et al., 2018; Fabricio et al., 2020; Ma and Chan, 2020).

### Putative Role of miRNAs in Cognitive Frailty

As regards the possible role of miRNAs in the pathogenesis of frailty with cognitive impairment and/or their potential use as biomarkers, to date, no studies are available considering cognitive frailty as a single condition. Furthermore, as reported above, there are only two studies analyzing changes in blood miRNAs specifically in frail subjects, while more evidence has been collected regarding cognitive impairment.

Although the limited information available makes it hard to depict a comprehensive picture of possible common miRNAs involved in both frailty and cognitive impairment, our review effort identified two miRNAs which were reported to be both differentially expressed in frail people and associated with cognitive deficits: miR-92a-3p and miR-532-5p (Table 1). Mature miR-92a-3p belongs to miR-17-92 cluster, located on chromosome 13 in the human genome. The miR-17-92 cluster, containing six miRNA precursors (miR-17, miR-18a, miR-19a, miR-20a, miR-19b-1, and miR-92a), is highly conserved among vertebrates and has fundamental roles during development (Concepcion et al., 2012; Mogilyansky and Rigoutsos, 2013). miR-92a-3p is a synaptic-related miRNA (Siedlecki-Wullich et al., 2021), involved in neural cells proliferation, differentiation, and maturation (Zhang et al., 2013; Xia et al., 2020). Intriguingly, it has been recently identified as a peripheral biomarker in different diseases, among which systemic lupus erythematosus (Kim et al., 2016), schizophrenia (Ma et al., 2018), and amyotrophic lateral sclerosis (Joilin et al., 2020). Moreover, miR-92a-3p was reported to increase ROS in mice (Gou et al., 2018), to regulates cartilage development and homeostasis (Mao et al., 2018), to participate in age-related pathophysiological processes including atherosclerosis and lipid metabolism (Loyer et al., 2014), cerebral white matter impairment (He et al., 2017), and cancer (Reis et al., 2020; Wang et al., 2021).

Mature miR-532-5p derived from pre-miR-532 which is localized on chromosome X in the human genome. miR-532-5p showed a neuroprotective effect reducing apoptosis, ROS production, and inflammation in cerebral ischemia-reperfusion injury (Shi et al., 2021b), and ischemic stroke (Mu et al., 2020). Moreover it has been implicated in inflammation (Yan et al., 2020), osteoporosis (Guo et al., 2020), as well as in tumor progression (Kim et al., 2021; Yu et al., 2021).

## Conclusion

In this review, we explored the possible use of miRNAs as both potential biomarkers and molecular effectors of frailty and cognitive impairment. We discussed the evidence linking changes in circulating miRNAs expression with these clinical conditions, with the final aim of shedding light on miRNAs that might be associated with cognitive frailty.

One of the limits of this study is that evidence giving a clear mechanistic link between frailty (or cognitive impairment) and miRNAs is still missing. Moreover. to date, only two works analyzed miRNAs expression in the plasma of frail patients, as potential peripheral biomarkers of frailty (Ipson et al., 2018; Rusanova et al., 2018). No further studies have been performed to evaluate the molecular mechanisms leading to changes in miRNAs expression in frail subjects, nor analyzing a possible involvement of these miRNAs in frailty etiopathogenesis. The same could be stated for studies linking miRNAs with cognitive impairment. Nevertheless, some of the miRNAs found to be differentially expressed in the blood of frail or cognitively impaired subjects have been reported to play a key role in cellular mechanisms associated with frailty and cognitive deficits, such as cellular senescence, oxidative stress, mitochondrial dysfunction, or inflammation (Thounaojam et al., 2013; Bigagli et al., 2016; Bu et al., 2017; Suh, 2018; Tahamtan et al., 2018; Konovalova et al., 2019; Sarkar et al., 2019; Slota and Booth, 2019; Catanesi et al., 2020). This suggests that miRNAs could be considered more than peripheral biomarkers, fostering the idea that miRNAs could be mechanistically involved in the etiogenesis of both frailty and cognitive impairment.

In this context, although more studies are needed, existing literature may suggest a potential use of iR-92a-3p and miR-532-5p not only as biomarkers of cognitive frailty, but also as in the context of the study of molecular mechanisms of frailty and cognitive diseases. Besides miR-92a-3p and miR-532-5p, other miRNAs consistently implicated in cellular mechanisms underlying both frailty and cognitive dysfunction, such for instance inflamma-miRs, SA-miRs, and miRNAs regulating oxidative processes, could have potential as biomarkers and molecular effectors of cognitive frailty as well.

In conclusion, although many works have proposed miRNAs as biomarkers of frailty and cognitive decline, the study of differentially expressed miRNAs in frailty is at its infancy, and reports on cognitive frailty are still missing. The identification of selected miRNAs differentially modulated in cognitive frailty could pave the way for innovative diagnostic and prognostic strategies, which may help the clinical management of people suffering from this condition, improving their life expectancy and quality of life. Furthermore, the study of miRNAs involvement in etiological mechanisms of cognitive frailty represents a promising tool for the identification of new targets for the development of novel therapeutic approaches, thus modeling health trajectories toward positive outcomes.

## Author Contributions

GC, LM, and AB: conceptualization. GC, LM, NV, and AB: writing—original draft. GC, LM, FB, AC, CF, AI, SM, MP, NV, and AB: writing—review and editing. All authors contributed to the article and approved the submitted version.

## Conflict of Interest

The authors declare that the research was conducted in the absence of any commercial or financial relationships that could be construed as a potential conflict of interest.

## Publisher’s Note

All claims expressed in this article are solely those of the authors and do not necessarily represent those of their affiliated organizations, or those of the publisher, the editors and the reviewers. Any product that may be evaluated in this article, or claim that may be made by its manufacturer, is not guaranteed or endorsed by the publisher.
